# Valproate Inhibits Methamphetamine Induced Hyperactivity via Glycogen Synthase Kinase 3β Signaling in the Nucleus Accumbens Core

**DOI:** 10.1371/journal.pone.0128068

**Published:** 2015-06-01

**Authors:** Bo Xing, Xiao-ping Liang, Peng Liu, Yan Zhao, Zheng Chu, Yong-hui Dang

**Affiliations:** 1 Department of Forensic Medicine, Key Laboratory of Environment and Genes Related to Diseases of Ministry of Education, Xi'an Jiaotong University School of Medicine, Xi'an, Shaanxi, PR China; 2 Xi’an Mental Health Center, Xi’an, Shaanxi, PR China; 3 Department of Pathophysiology, School of basic Medicine Sciences, Xi'an Medical University, Xi'an, Shaanxi, PR China; Chiba University Center for Forensic Mental Health, JAPAN

## Abstract

Valproate (VPA) has recently been shown to influence the behavioral effects of psycho-stimulants. Although glycogen synthase kinase 3β (GSK3β) signaling in the nucleus accumbens (NAc) plays a key role in mediating dopamine (DA)-dependent behaviors, there is less direct evidence that how VPA acts on the GSK3β signaling in the functionally distinct sub-regions of the NAc, the NAc core (NAcC) and the NAc shell (NAcSh), during psycho-stimulant-induced hyperactivity. In the present study, we applied locomotion test after acute methamphetamine (MA) (2 mg/kg) injection to identify the locomotor activity of rats received repeated VPA (300 mg/kg) pretreatment. We next measured phosphor-GSK3β at serine 9 and total GSK3β levels in NAcC and NAcSh respectively to determine the relationship between the effect of VPA on MA-induced hyperlocomotor and changes in GSK3β activity. We further investigated whether microinjection of VPA (300 μg/0.5 μl/side, once daily for 7 consecutive days) into NAcC or NAcSh could affect hyperactivity induced by MA. Our data indicated that repeated VPA treatment attenuated MA-induced hyperlocomotor, and the effect was associated with decreased levels of phosphorylated GSK3β at Ser 9 in the NAcC. Moreover, repeated bilateral intra-NAcC, but not intra-NAcSh VPA treatment, significantly attenuated MA-induced hyperactivity. Our results suggested that GSK3β activity in NAcC contributes to the inhibitory effects of VPA on MA-induced hyperactivity.

## Introduction

Bipolar disorder is a severe mental illness that affects approximately 1% of the world’s population [1]. It is characterized by mood swings of mania and depression. In the depressed cycle, individuals can have all or any of the symptoms of depressive disorders. In the manic cycle, the sufferers may be overactive, over talkative, and have a great deal of energy. Although valproate (VPA) has been used in the clinic for many years as mood stabilizer for bipolar disorder, the molecular mechanisms by which it exert therapeutic effects have not been fully established.

Acute methamphetamine (MA) intoxication are reported to be associated with euphoria, talkativeness, and psychomotor agitation that can resemble the manic or mixed phase of bipolar disorder [2]. The pharmacological animal models of bipolar disorder to date involves the use of single high dose of MA to mimic the manic symptoms observed in human [3]. Locomotor hyperactivity induced by psycho-stimulants results from facilitation of dopaminergic transmission in the ventral striatum [4,5], and this behavioral response can be attenuated by administration of VPA into nucleus accumbens (NAc) [6]. However, the inhibitory effect of VPA on hyperlocomotor activity induced by psycho-stimulants could involve changes in functionally distinct sub-regions of the NAc, the NAc core (NAcC) and the NAc shell (NAcSh). Identification of the anatomical locus and underlying mechanisms will provide insight into the pathogenesis of mania, and may shed some light on providing novel strategies for the prevention or treatment of mania.

According to their distinct histochemical characteristics and hodological organization, the NAcC and NAcSh have been distinguished in the ventral striatum. Traditionally, the NAcC has been involved in motor functions, similar to the dorsal striatum, whereas NAcSh has been seen as the transition zone between the striatum and the extended amygdala, participating in the emotional and motivational processing [7]. Although NAc as a whole has been well documented in psycho-stimulant-related behaviors, considerably less attention has been paid to the specific roles of its two sub-regions.

Glycogen synthase kinase 3 (GSK3) is a multifunctional serine/threonine kinase that was originally identified as a regulator of glycogen metabolism [8]. In mammals, there are two closely related isoforms, GSKα and GSK3β, are present [9]. Only the latter isoform, however, is highly enriched in the brain [10,11], where it has been implicated in the pathology of several neuropsychiatric diseases including Alzheimer’s disease, schizophrenia, and bipolar disorder [12,13]. Unlike most kinases, GSK3 is constitutively active under basal conditions, and most pathways, such as Akt/protein kinase B signaling and Wnt signaling, converging on GSK3 decrease its activity. Phosphorylation, especially at Ser 9, is the major mechanism inhibiting GSK3β activity, whereas the expression levels of GSK3β are generally stable. Recently, several lines of study have demonstrated GSK3β is required for dopaminergic signaling and relate behaviors [14]. For instance, inhibitors of GSK3 have been shown to attenuate cocaine- and amphetamine-induced hyperactivity, behavioral sensitization, and conditioned reward [15–18]. Hyper-locomotion in DA transporter knockout mice can be blocked by GSK3 inhibitors, while GSK3 heterozygote mice have a blunted response to amphetamine-induced locomotor activity [19].

In this study, we investigated the effects of systemic repeated administration of VPA on MA-induced locomotor activity in rats. We then further measured the changes of levels of total GSK3β and phosphorylated GSK3β at serine 9 (**a major inhibitory form of GSK3β**) in the NAcC and NAcSh after behavioral tests among groups. At last, we examined the effects of repeatedly microinjected VPA into the NAcC and NAcSh on MA-induced hyper-locomotor activity. Our data suggest that VAP attenuated the MA related hyperactivity, at least in part, through restoring GSK3β signaling in NAcC.

## Materials and Methods

### Animals

Male Sprague–Dawley rats weighing 200–250 g on arrival were obtained from the Medical Experimental Animal Center of Xi’an Jiaotong University (Shaanxi, PR China). They were housed four each cage under standard conditions of temperature (22 ± 1°C) and a 12:12 h light/dark cycle with food and water *ad libitum*. This study was carried out in strict accordance with the recommendations in the Guide for the Care and Use of Laboratory Animals of the National Institutes of Health. The protocol was approved by the Committee on the Ethics of Animal Experiments of Xi’an Jiaotong University (Permit Number: 2009086). All surgery was performed under sodium pentobarbital anesthesia, and all efforts were made to minimize animal suffering and to reduce the number of animals used.

### Surgery

Rats were anesthetized with sodium pentobarbital (50mg/kg, intraperitoneally) and the head was immobilized in a stereotaxic frame. The stainless steel guide cannulae (23 gauge) were implanted bilaterally 2.0 mm just above the NAcC or NAcSh as reported previously [20], at the following coordinates: for NAcC, 1.32 mm anterior to bregma, ± 2.0 mm lateral, 5.8 mm ventral to brain surface; for NAcSh, 1.56 mm anterior to bregma, ± 0.8 mm lateral, 6.5 mm ventral to brain surface [21]. Stylets were inserted into the guide cannulae to prevent occlusion and all rats were treated with sodium penicillin (0.2 million U/day for 5 days, intraperitoneal injection) after surgery to prevent intracerebral infection. The rats were carefully nursed and housed one per cage, allowed to recover for 5–7 days. Rats received intraperitoneal injection of VPA and corresponding saline did not undergo surgery.

### Drugs and pharmacological procedures

**Fig 1:**
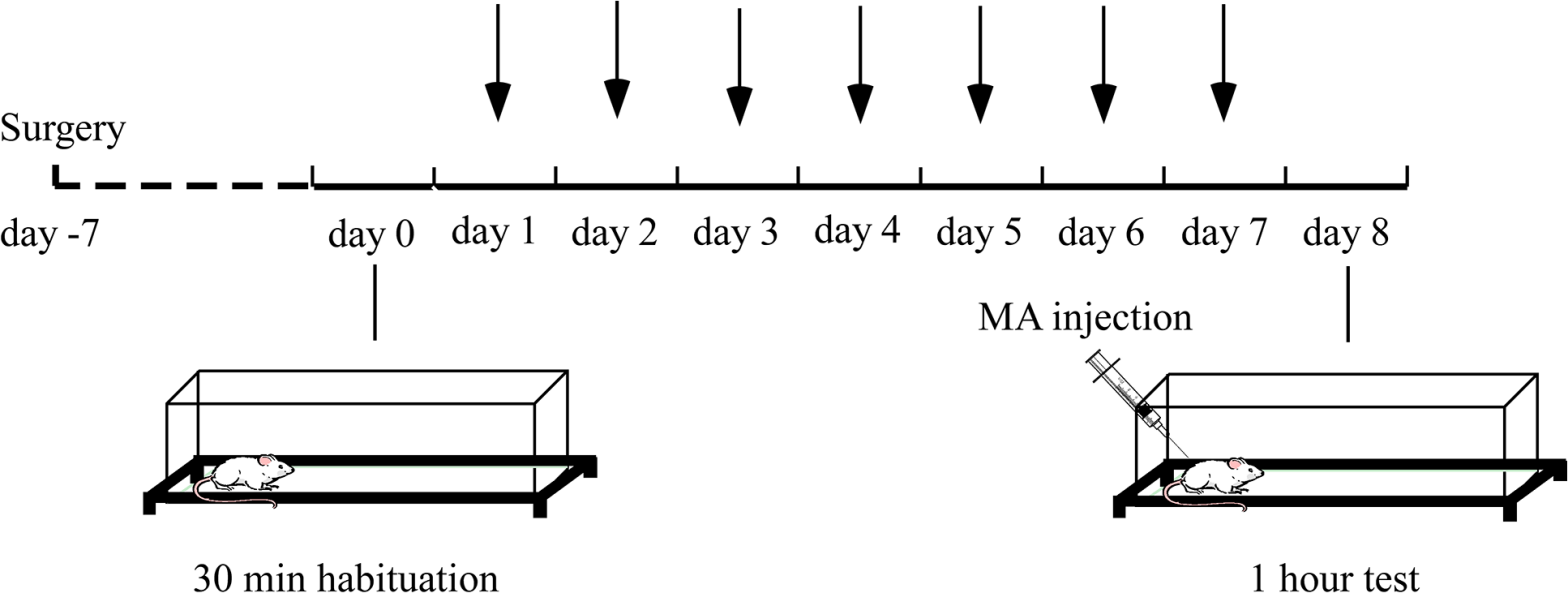
Schematic frame of the drug treatment and behavioral procedure. The black arrows indicate the seven consecutive days VPA pre-treatment (either 300 mg/kg, intraperitoneal injection or 300μg/0.5μl/side, intracranial injection) or saline controls before the MA injection and locomotor test.

D-methamphetamine hydrochloride (National Institute for the Control of Pharmaceutical and Biological Products, Beijing, PR China) and sodium valproate (Sigma Chemical, St. Louis, MO, USA) was dissolved in sterile saline (0.9% sodium chloride). As shown in Fig 1, animals were treated for experimental procedures as following: on day 0, rats were first habituated to the locomotor activity boxes for 30 min and returned to home cages. From day 1 through day 7, intraperitoneal injection (300 mg/kg) or bilateral microinjections (300μg/0.5μl/side)of VPA were made at home cages once a day. The doses of VPA were based upon previous studies [22,23]. On day 8, they were all habituated again to the locomotor activity boxes for 30 min followed by either saline or single MA (2 mg/kg, intraperitoneal injection) injection, then immediately returned to the test boxes and their horizontal locomotion measured for 1 hour. During the microinjection, the rats were gently held and the stylets was removed and replaced with injector cannula. Injector cannula extended an additional 2.0 mm below the guide cannula. VPA was slowly infused through a 1.0 μl Hamilton syringe over 1 min with the injector cannula remaining in the guide cannula for another minute to prevent backflow. Drug solutions were freshly prepared before each experiment. There were four groups included in each microinjection (NAcC or NAcSh) experiment: SAL microinjections with MA challenge (SAL-MA), SAL microinjections without MA challenge (SAL-SAL), VPA microinjections with MA challenge (VPA-MA), and VPA microinjections without MA challenge (VPA-SAL).

### Locomotor activity test

The test room was dimly lit with indirect white lighting. Locomotor activity was assessed using an apparatus consisted of a square Plexiglas box of 45 cm×45 cm and 45 cm high. The distance that each rats moved was determined by a video-computerized tracking system (SMART, Panlab SL, Barcelona, Spain).

### Tissue sample preparation and western blot

Rats without surgery were sacrificed by cervical dislocation immediately after locomotor activity test. As described previously [16], the brains were rapidly removed and snap-frozen on dry ice. The NAcC and NAcSh punches were obtained with a 1 mm punch tool (homemade by 16-gauge needles) from 400-mm-thick coronal sections taken on a sliding freezing microtome according to the rat brain atlas [21]. The samples were homogenized in ice-cold RIPA buffer (50 mM Tris, pH 8.0, 1% Triton X-100, 0.1% SDS, 150 mM NaCl) containing protease inhibitor and phosphatase inhibitor cocktail tablets (Roche Applied Science, Mannhelm, Germany). The homogenate was incubated on ice for 20 min and centrifuged at 13,000 *g* for 20 min at 4°C. Supernatants were collected and protein concentrations were determined by Bradford BCA protein assay and stored at -80°C until further use. For western blot, samples treated with β-mercaptoethanol were denatured at 95°C for 5 min, then 10 μg of extracts were subjected to 12% SDS-PAGE, subsequently blotted onto a nitrocellulose membrane (Millipore, Bedford, MA, USA). After blocking with 5% non-fat milk in 1 × Tris-buffered saline with 0.1% Tween-20 (TBST), pH 7.4, blots were probed with primary antibodies followed by appropriate secondary antibodies. Signals were detected by enhanced chemiluminescence (Pierce Biotechnology, Rockford, IL, USA) and quantified by a Quantity One program (Bio-rad, Hercules, CA, USA). We used primary antibodies against phosphor-GSK3β (Ser 9), GSK3β (Cell Signaling, MA, USA) at 1:5000 dilutions, and goat anti-rabbit lgG horseradish peroxidase (HRP)-conjugated secondary antibody (Bioworld, CA, USA) at 1:10,000 dilution. The β-actin (Cell Signaling, MA, USA, at 1:10,000) was used as an internal control. The results were analyzed using densitometry. Ratios of phosphor- to total GSK3β and total GSK3β to β-actin were calculated respectively for each sample. Saline group was set at 1. All western blots were conducted at least three times to ensure reproducibility.

### Histology

As previously reported [24],after locomotor activity test, the microinjection sites of rats with surgery were marked by injecting Pontamine Sky Blue dye injection (0.5 μl, 2% in 0.5 M sodium acetate solution). Under deeply anesthetized with chloral hydrate (40 mg/kg), the rats were perfused transcardially with 0.01 M phosphate-buffered saline (PBS, pH 7.4), followed by 10% formalin. The brains were then removed and post-fixed in 10% formalin solution for 3–7 days. Coronal sections from the injection site were obtained at 30 μm using a freezing microtome. The brain slices were processed for cresyl violet staining according to standard Nissl-staining procedures. The injection sites were plotted on the coronal sections modified from the Paxinos and Watson atlas [21]. The injection sites were histologically identified to be within the NAcC or NAcSh for data analysis (S1 Fig).

### Data analysis

All data were expressed as mean ± S.E.M. The effects on locomotion activity test and western blot were analyzed using one-way analyses of variance (ANOVA). Where appropriate, Bonferroni post hoc tests were used to determine group differences. Comparisons were considered statistically significant at *p* < 0.05.

## Results

### Repeated systemic VPA treatment inhibited MA-induced hyper-locomotor activity

**Fig 2:**
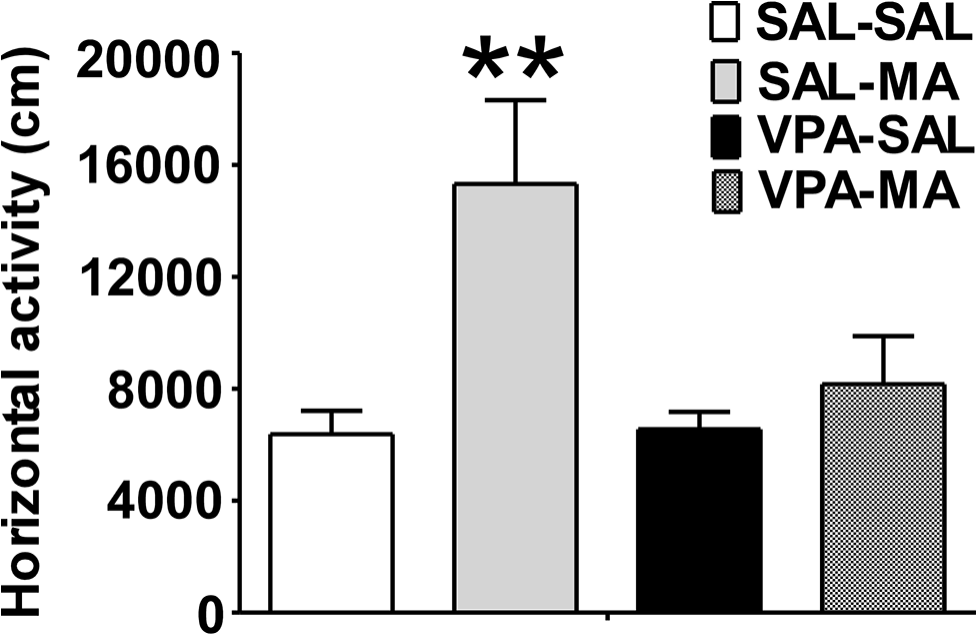
Effect of pre-treatment with VPA on MA-induced hyper-locomotor activity. Repeated pre-treatment of VPA (300 mg/kg, seven consecutive days, intraperitoneal once a day) significantly attenuates the effect of MA injection (2 mg/kg, intraperitoneal) on locomotor activity compared with saline (SAL) controls. Data are expressed as mean ± SEM, n = 8 per group, ** *p* < 0.01 versus SAL-SAL, one-way ANOVA followed by Bonferroni's Multiple Comparison Test.

In the first experiment, we examined the effects of repeated systemic administration of VPA on locomotor activity after single MA or saline injection (Fig 2). After 7 days VPA or saline pre-treatment, the rats received MA or saline injection prior to locomotor test. There were significant differences among groups in the total distance travelled during the 1-hour locomotion test (*F*_(3, 31)_ = 5.483, *p* = 0.0043). As expected, single MA injection resulted in a significantly increased horizontal locomotion compared to controls given saline (SAL-MA vs. SAL-SAL, 15322 ±2997 vs 6381 ± 834.2, *p* < 0.01, Bonferroni *post hoc* test). The hyper-locomotor induced by MA were significantly inhibited by a 7-day VPA pre-treatment (SAL-MA vs. VPA-MA, *p* < 0.05, 15322 ±2997 vs 8170 ± 1713, Bonferroni *post hoc* test), which did not affect basal activity (VPA-MA vs. VPA-SAL, 8170 ± 1713 vs 6557 ± 621.3, *p* > 0.05, Bonferroni *post hoc* test). These results showed that repeated VPA administration inhibited hyper-locomotion caused by single MA treatment.

### Repeated systemic VPA treatment restored MA-induced decreased phosphor-GSK3β at Ser 9 levels in NAcC

**Fig 3:**
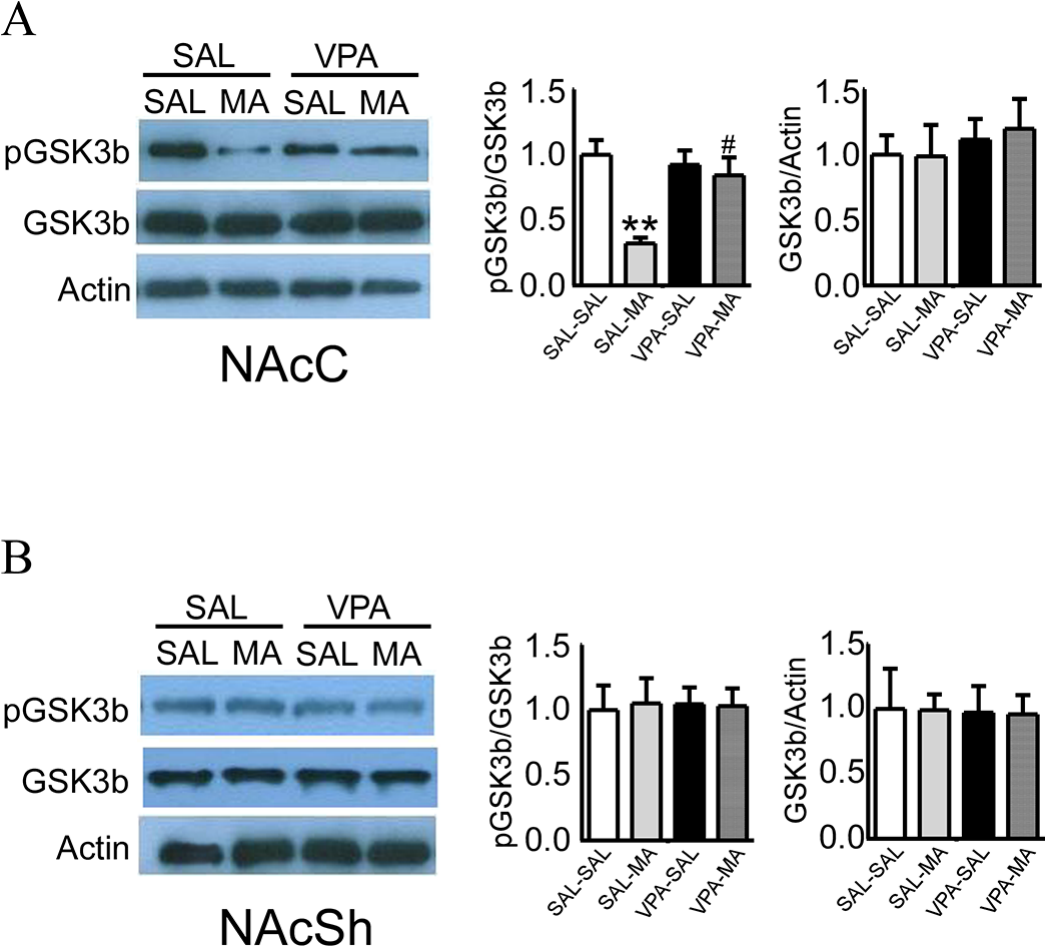
Effect of pre-treatment with VPA on MA-induced activation of GSK3β signaling in NAcC and NAcSh. Representative blots and summary histograms of phospho-GSK3β at Ser 9 (pGSK3b) and total GSK3β (GSK3b) from NAcC (A) and NAcSh (B) tissues from repeated saline (SAL) and VPA pre-treatment rats which then received SAL or MA injection. SAL-SAL group levels were set at 1 for quantifications. Data were expressed as mean ± SEM, n = 8 per group, ** *p* < 0.01 versus SAL-SAL, ^#^ *p* < 0.05 versus SAL-MA, one-way ANOVA followed by Bonferroni's Multiple Comparison Test.

To determine the role of GSK3β activity in NAc sub-regions during the locomotor hyperactivity induced by MA injection, we examined the levels of phosphor-GSK3β at Ser 9 and total GSK3β by western blot in NAcC and NAcSh respectively after the behavioral tests. Analysis by ANOVA revealed significant differences of levels of phosphor-GSK3β (Ser 9) (ratio of pGSK3β:GSK3β) in the NAcC (*F*_(3, 27)_ = 8.298, *p* < 0.001), but not NAcSh (*F*_(3, 27)_ = 0.201, *p* = 0.8948) among groups. As shown in Fig 3, rats given saline pretreatment followed by a MA injection showed significantly lower levels of phosphor-GSK3β at Ser9 in NAcC compared to that followed by saline injection (SAL-MA vs. SAL-SAL, 0.32 ± 0.04 vs 1.00 ± 0.11, *p* < 0.01, Bonferroni *post hoc* test). Although VPA pretreatment did not alter the GSK3β activity with saline injection (SAL-SAL vs. VPA-SAL, 1.00 ± 0.11 vs 0.92 ± 0.11, *p* > 0.05, Bonferroni *post hoc* test), it did reverse the decreased levels of phosphor-GSK3β (Ser 9) caused by MA challenge (VPA-MA vs. SAL-MA, 0.84 ± 0.14 vs 0.32 ± 0.04, *p* < 0.05, Bonferroni *post hoc* test). Total GSK-3β levels were not affected by VPA pretreatment or MA injection in the NAc core (*F*_(3, 27)_ = 0.2557, *p* = 0.8565) or shell (*F*_(3, 27)_ = 0.0081, *p* = 0.9990).

### Repeated microinjections of VPA into NAcC, but not NAcSh, inhibited MA induced hyper-locomotion

**Fig 4:**
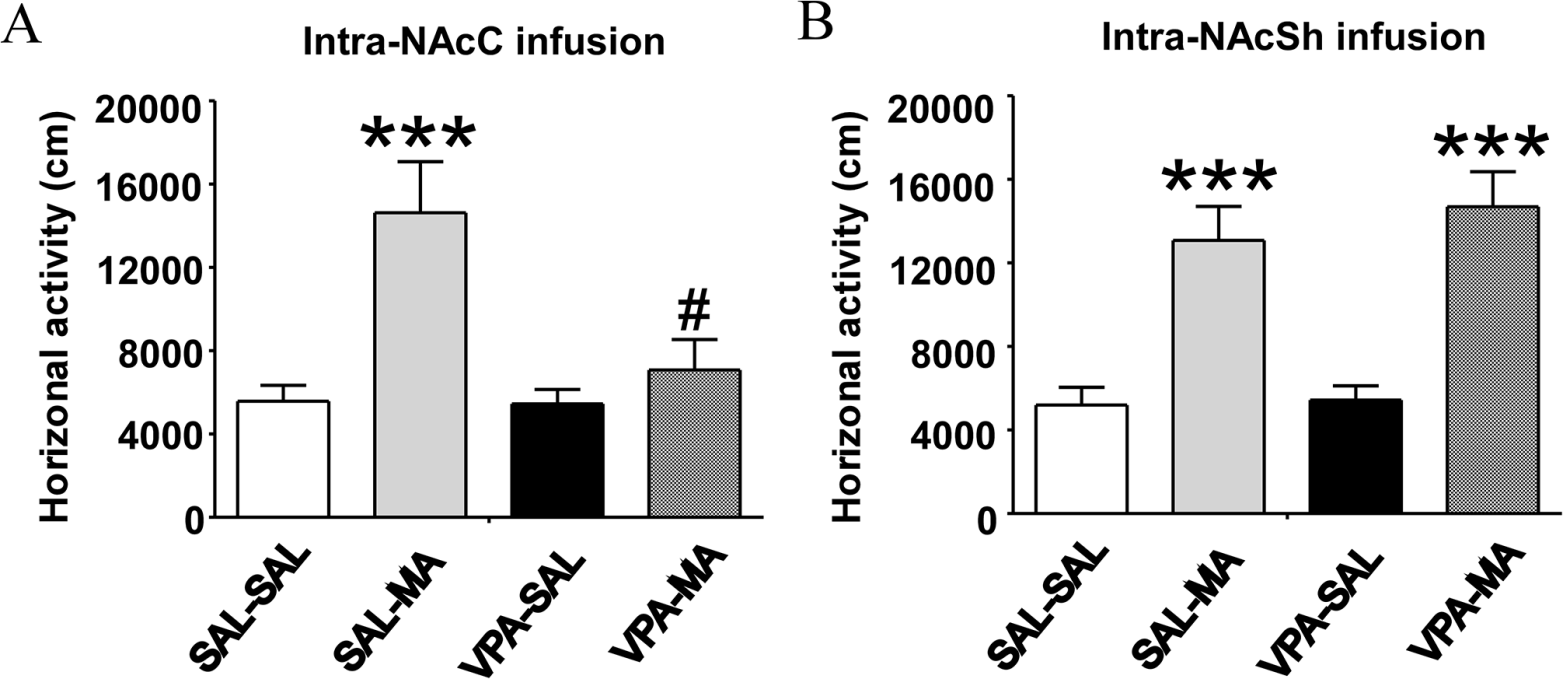
Effects of repeated microinjections of VPA into NAcC and NAcSh on MA-induced hyper-locomotor activity. Repeated pre-treatment of VPA (300μg/0.5μl/side, consecutive seven days, intracranial injection once a day) into NAcC (A) but not NAcSh (B) significantly attenuates the effect of MA injection (2 mg/kg, intraperitoneal) on locomotor activity compared with saline (SAL) controls. MA induced hyper-locomoter in rats with SAL infusion in both NAcC and NAcSh infusions (SAL_NAcC-MA_ vs. SAL_NAcC-SAL_, *p* < 0.05; SAL_NAcSh-MA_ vs. VPA_NAcSh-SAL_, *p* < 0.05 Bonferroni post hoc test). VPA did not affect locomotor activity without MA administration (SAL_NAcC-SAL_ vs. SAL_NAcC-SAL_, *p* > 0.05; SAL_NAcSh-SAL_ vs. VPA_NAcSh-SAL_, *p* > 0.05 Bonferroni post hoc test). VPA infusions in NAcC but not NAcSh caused inhibitory effect on MA induced hyper-locomotion compared with saline controls (SAL_NAcC-MA_ vs. VPA_NAcC-MA_, *p* < 0.05; SAL_NAcSh-MA_ vs. VPA_NAcSh-MA_, *p* > 0.05 Bonferroni post hoc test). However, there was no significant difference between rats given VPA infusions in NAcSh and saline controls (SAL_NAcSh_-MA vs. VPA_NAcSh-MA_, *p* > 0.05, Bonferroni post hoc test). Data are expressed as mean ± SEM, n = 8 per group, *** *p* < 0.001 versus SAL-SAL, ^#^ *p* < 0.05 versus SAL-MA, one-way ANOVA followed by Bonferroni's Multiple Comparison Test.

To determine the roles of NAc sub-regions in the effect of VPA on MA-induced hyper-locomotion, we repeatedly microinjected VPA into NAcC or NAcSh once a day for 7 days before the rats received acute MA injection and following locomotor test. As shown in Fig 4, There were significant differences among groups in the total distance travelled during the 1-hour locomotion test for intra-NAcC treatment (*F*_(3, 31)_ = 8.270, *p* = 0.0004) or intra-NAcSh treatment (*F*_(3, 31)_ = 14.97, *p* < 0.0001). As indicated by *post hoc* analysis, VPA infusions in NAcC caused inhibitory effect on MA related hyper-locomotion compared with saline controls, (SAL_NAcC_-MA vs. VPA_NAcC_-MA, 14628 ± 2450 vs 7075 ± 1460, *p* < 0.05, Bonferroni *post hoc* test). However, there was no significant difference between rats given VPA infusions in NAcSh and saline controls (SAL_NAcSh_-MA vs. VPA_NAcSh_-MA, 13077 ± 1629 vs 14687 ± 1680, *p* > 0.05, Bonferroni *post hoc* test). These results suggested that VPA acting on NAcC, but not NAcSh, play a critical role in inhibiting the hyper-locomotor activity induced by MA.

## Discussion

Results from the present study demonstrated that systemic pretreatment of the mood stabilizer VPA significantly inhibited MA-induced hyper-locomotion. The effect was associated with decreased levels of phosphorylated GSK3β at Ser9 in the NAcC but not NAcSh. Furthermore, the effect of systematic VPA treatment could be mimicked by microinjection of the VPA directly into the NAcC, whereas intra-NAcSh infusions have no effect on the hyper-locomotion induced by MA.

It is generally accepted that striatum plays a central role in the neural circuits underlying behavioral characteristics relevant to psychomotor stimulants. DA present in the region of the NAc contributes to the increase in locomotor activity induced by MA, while stereotypy is mediated by dorsal striatal DA (Taylor and Robbins, 1984). DA modulates movement, reward, cognition, and emotion through activation of dopamine G protein–coupled receptors that belong to two subclasses, the D1 receptor (D1R and D5R) class and the D2 receptor (D2R, D3R, and D4R) class, their highest expression being in the striatum (for review see [25]). Traditionally, DA modulation of neuronal and synaptic activities is known to be associated with the G-protein–dependent cyclic AMP-protein kinase A-dopamine, cyclic AMP-regulated phosphoprotein of 32 kDa (cAMP-PKA-DARPP32) pathway, which is activated by D1R and inhibited by D2R stimulation [26]. Emerging evidence suggests that D2R also activate GSK3β signaling to affect DA-dependent behaviors and the actions of dopaminergic psychostimulants and antipsychotics [27]. Indeed, we found that GSK3β signaling is involved for hyper-dopaminergic induced by MA in the rat NAc. Furthermore, previous studies suggested that GSK-3β activity in the NAc mediates the initiation and expression of cocaine-, amphetamine- and MA-induced locomotor sensitization and reward conditioning [15–18,28].

While NAc are clearly important for hyper-dopamine related behaviors, its two sub-regions, NAcC and NAcSh, play different roles in addiction [29,30]. There are increasing evidence that differences between the NAcC and NAcSh in modulating DA-related behaviors [31–33], the underlying mechanisms remain incomplete. To investigate whether the GSK3β signaling in NAcC and NAcSh are distinctly regulated by MA, we compared the GSK3β activity in these regions after single MA injection. Our data revealed a significant difference in phosphor-GSK3β at Ser 9 levels between NAcC and NAcSh, with no change of the total GSK3β levels in these regions. These results indicate that GSK3β activity are differentially recruited in NAcC and NAcSh by MA. Microdialysis studies in animals have shown that different psycho-stimulants have distinct effects on increasing extracellular DA in NAcC and NAcSh [34,35]. In our case, the extracellular dopamine should increase extracellular dopamine in the shell and in the core to a similar extent because of the high dose used (2 mg/kg). Notably, previous studies have suggested differential roles of the NAcC and NAcSh in the effects of psychostimulants and motivated behavior [16,28,36]. Behavioral sensitization to MA is associated with an increase in GSK3β activity in the NAcC [28]. Accordingly, systemic or regional blockage of GSK3β in NAcC but not NAcSh attenuated MA-induced behavioral sensitization [28]. It is speculated that locomotor activity results from acute MA administration mainly exerted on NAcC by postsynaptic mechanisms. Our data showed that the D2R mediated GSK3β signaling pathway could be one of the possible underlying molecular events differentially associated with NAcC and NAcSh after acute MA treatment. Importantly, although GSK3β could act on presynaptic neurotransmission [37], its action probably though postsynaptic mechanisms. Specifically, GSK3β presented in the vicinity of synapses could interplay with α-amino-3-hydroxy-5-methyl-4-isoxazole propionic acid (AMPA) receptor, resulting in insertion of AMPA receptor [38]. Moreover, the GSK3β was a key regulator in the phosphorylation of PSD-95, a major scaffold protein in the postsynaptic density (PSD) of excitatory synapses, leading to AMPA receptor mobilization [39]. Further studies are required to determine the precise mechanism of the involvement of GSK3β in NAcC exposed to psychomotor stimulants.

VPA is one of the well-known mood stabilizers in the pharmacotherapy of bipolar disorder, while the therapeutic target is still being identified. Recently, its inhibitory effect on GSK3 has received a great deal of attention. Several studies have shown that VPA, as well as specific GSK3 inhibitors such as SB216763, inhibits psycho-stimulant caused hyperactivity and sensitization [15–18,28]. In a previous study, acute amphetamine induced hyperactivity could be inhibited by VPA, and this effect was associated with the ability of VPA to increase GSK3 activity (indicated by decreased inhibitory pGSK3β at Ser 9) in the caudate putamen and frontal cortex without changes in NAc [17]. However, our results have indicated that the VPA decreases the MA induced hyper-locomotor activity associated with increasing GSK3β activity (indicated by decreased inhibitory pGSK3β at Ser 9) in NAcC. The inconsistent results have reflected that the changes of GSK3β activity after acute MA treatment are mainly within the core sub-region of NAc. To support this explanation, the same group recently have shown that inhibitory effect of VPA on acute cocaine induced hyper-locomotion is associated with reduced pGSK3β at ser 9 levels in NAcC but not NAcSh [18]. Although previous study have revealed that repeated treatment of VPA differentially modulates DA dependent behaviors induced by amphetamine and cocaine [40], our data further extended these findings by indicating the directly microinjecting VPA into NAcC, similar to systemic VPA treatment, blocks the MA-induce hyperactivity.

In addition to inhibition of GSK3, VPA probably reduces the hyper-locomotion caused by MA through modulation of γ-aminobutyric acid (GABA) transmission in NAcC. As a well-known GABA enhancer, VPA increases GABAergic transmission by increasing availability of synaptic GABA or enhance postsynaptic GABA action [41]. Previous studies have suggested that central GABAergic system is a key regulator of the activity of DA release and dopaminergic activity [42]. The agonists of GABA_A_ as well as GABA_B_ receptors can block the hyper-locomotor activity and stereotype behaviors resulting from hyper-dopaminergic conditions [43,44]. Notably, a recent study has shown that activation of GABA receptors inhibits protein kinase B (Akt), resulting in upregulating of GSK3 signaling in a β-arrestin-dependent pathway [45], indicating a possible interplay between GABA transmission and GSK3 activity both of which could be regulated by VPA.

In summary, the hyperactivity induced by MA is associated with GSK3β activity in NAcC, but not NAcSh. As such, microinjection of VPA into NAcC can effectively mimic the inhibitory effect of repeated systemic treatment of VPA on MA induced hyper-locomotion. Our current results emphasize the importance of sub region-specific modulation of GSK3β signaling within NAc in the control of DA-dependent behaviors.

## Supporting Information

S1 Fig The schematic figures of injection sites verification within NAcC (A) and NAcSh (B). Black dots show the location of the tips of the injection cannula for the rats. The figures are adapted from diagrams of the stereotaxic atlas of the rat brain (The Rat Brain in Stereotaxic Coordinates, 2004, the fifth edition).
(TIF)
