# Supplementary figures and images for: Valproate Inhibits Methamphetamine Induced Hyperactivity via Glycogen Synthase Kinase 3β Signaling in the Nucleus Accumbens Core
Source: PLoS One. 2015 Jun 1;10(6):e0128068. doi: 10.1371/journal.pone.0128068 (PMC4452337; doi:10.1371/journal.pone.0128068)

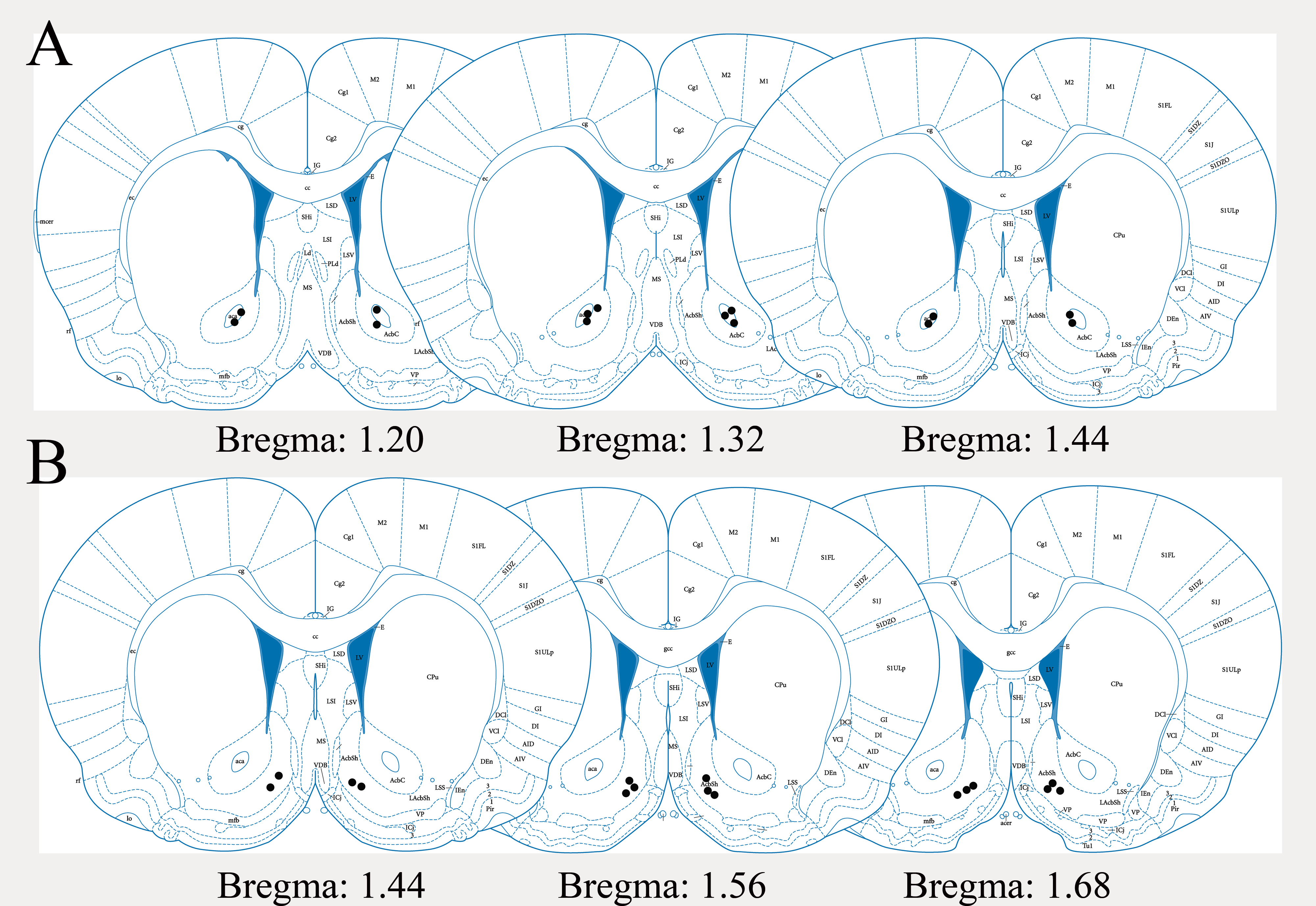

Supplement: S1 Fig — Black dots show the location of the tips of the injection cannula for the rats. The figures are adapted from diagrams of the stereotaxic atlas of the rat brain (The Rat Brain in Stereotaxic Coordinates, 2004, the fifth edition). (TIF) [file pone.0128068.s001.tif]
